# Correlations between Properties of Pore-Filling Ion Exchange Membranes and Performance of a Reverse Electrodialysis Stack for High Power Density

**DOI:** 10.3390/membranes11080609

**Published:** 2021-08-10

**Authors:** Hanki Kim, Jiyeon Choi, Namjo Jeong, Yeon-Gil Jung, Haeun Kim, Donghyun Kim, SeungCheol Yang

**Affiliations:** 1 Jeju Global Research Center, Korea Institute of Energy Research, Jeju-si 63357, Korea; hankikim@kier.re.kr (H.K.); jychoi@kier.re.kr (J.C.); njjeong@kier.re.kr (N.J.); 2 School of Materials Science and Engineering, Changwon National University, Changwon-si 51140, Korea; jungyg@changwon.ac.kr (Y.-G.J.); rlagkdms324@changwon.ac.kr (H.K.); 20165096@changwon.ac.kr (D.K.); 3 Department of Materials Convergence and System Engineering, Changwon National University, Changwon-si 51140, Korea

**Keywords:** ion exchange membrane, pore-filling, reverse electrodialysis, resistance, permselectivity

## Abstract

The reverse electrodialysis (RED) stack-harnessing salinity gradient power mainly consists of ion exchange membranes (IEMs). Among the various types of IEMs used in RED stacks, pore-filling ion exchange membranes (PIEMs) have been considered promising IEMs to improve the power density of RED stacks. The compositions of PIEMs affect the electrical resistance and permselectivity of PIEMs; however, their effect on the performance of large RED stacks have not yet been considered. In this study, PIEMs of various compositions with respect to the RED stack were adopted to evaluate the performance of the RED stack according to stack size (electrode area: 5 × 5 cm^2^ vs. 15 × 15 cm^2^). By increasing the stack size, the gross power per membrane area decreased despite the increase in gross power on a single RED stack. The electrical resistance of the PIEMs was the most important factor for enhancing the power production of the RED stack. Moreover, power production was less sensitive to permselectivities over 90%. By increasing the RED stack size, the contributions of non-ohmic resistances were significantly increased. Thus, we determined that reducing the salinity gradients across PIEMs by ion transport increased the non-ohmic resistance of large RED stacks. These results will aid in designing pilot-scale RED stacks.

## 1. Introduction

The salinity gradient power (SGP) is a remarkable renewable energy source that generates electrical power by mixing water with different salinities, such as seawater and river water. The SGP has tremendous energy potential of up to 2.4 TW on global estuaries [1]. There are several SGP engineering tools including pressure-retarded osmosis (PRO), which utilizes the movement of water through a water-permeable membrane; reverse electrodialysis (RED), which utilizes the movement of ions through an ion exchange membrane (IEM); and capacitive mixing (CapMix), which utilizes the adsorption and desorption of ions in materials such as activated carbon. Among these tools, CapMix is still in the early stages of research, resulting in low power density, whereas studies on PRO and RED have progressed to the pilot scale under natural conditions [2,3,4,5]. Specifically, RED is an electrochemical (or electro-membrane) process and has additional benefits as compared to PRO in terms of fouling resistance [6]. Fouling on IEMs in the RED stack can be addressed by adopting polarity reversal and air sparging methods [7]. Therefore, the pilot-scale RED stack could be continuously operated using municipal wastewater and seawater [8]. In addition, RED can be integrated and combined with technologies such as reverse osmosis [9], microbial fuel cell [10], redox flow battery [11], etc., so that its application can be further enlarged and its functions become more complete, which also makes the application based on RED technology and basic research gradually shift from traditional electricity production to new energy and environmental protection.

The RED stack comprises the repetition of unit cells, which consist of one cation exchange membrane (CEM), one anion exchange membrane (AEM), spacers, and gaskets. The redox species recirculate between the anode and cathode compartments to generate an electrical current following an appropriate electrochemical reaction. When the feed solutions (seawater and river water) are pumped into the flow channels, the cations and anions in seawater are selectively transported through the CEM and AEM, respectively. Ion flux through these membranes is converted to electrical current on the electrodes, and the intensity of the electrical current is mainly related to the electrical resistance of the IEMs. Thus, the development of high-performance IEMs is significant for the commercialization of the RED system.

The IEMs, selectively transporting specific ions, are a key component determining the performance of the RED. Therefore, many commercially available IEMs have been utilized and evaluated in the RED stack. Most commercial IEMs showed low performance in RED stack due to their high resistance [5,12,13]. To promote RED stack performance, the characteristics of IEMs including electrical resistance and permselectivity were continuously improved [5]. The low electrical resistance of IEM allows for high current density in the RED stack, while the high permselectivity allows for high electrical voltage, indicating the requirement of both characteristics in the same IEM. Previous studies focused on reducing membrane thicknesses for low electrical resistance [13] and increasing fixed charge density to improve permselectivity [14,15,16]. However, simultaneous achievement of low electrical resistance and high permselectivity is still a significant challenge for IEM development for commercial RED stacks. On the other hand, the challenging issue of IEM development is also related to the evaluation of IEM performance in the commercial (large size) RED stack. However, thus far, evaluations of RED-specific IEMs were generally performed using a laboratory-scale (small size) RED stack [17,18,19,20,21,22,23] because mass production of high-performance IEMs for RED stacks without an optimized roll-to-roll (R2R) process has been quite difficult [24,25]. Therefore, the evaluation and validation of large RED stacks is limited to the use of commercial IEMs [8,26,27,28,29,30]; furthermore, there are few studies on the correlation between IEM characteristics and large-scale RED performance although this is a critical factor in designing the strategy for upscaling RED. 

Notably, Moreno et al. investigated the upscaling strategy for RED with stack dimensions of 6 × 6 cm^2^, 10 × 10 cm^2^, 22 × 22 cm^2^, and 44 × 44 cm^2^, including 50 cell pairs. They revealed that the residence time of feed water in the stack, low water permeability, and high permselectivity of IEMs are important for designing large RED stacks [26]. When compared with the performance difference between the small and large RED stacks with the same linear velocity of feed water, the performance including the power density of the RED stack decreased with increasing RED stack size due to the corresponding salinity gradient reduction between high- and low-concentrated feed water. However, they did not consider the correlation factors between IEM characteristics and large RED stack performance. 

In the recent years, several studies were conducted to develop pore-filling ion exchange membranes (PIEMs) for high-performance RED stacks with high power density and/or high energy efficiency [13,24,25,31,32,33]. The PIEMs fabricated in our laboratory have low electrical resistance because of a thin porous substrate and conductive electrolyte in its nanopores. Moreover, the PIEMs have a competitive high permselectivity as compared with the commercial IEMs. Therefore, the RED stack with PIEMs presents 10–20% higher power density than the RED stack with commercial IEMs [34]. Although upscaling is also a critical issue for the RED stack with PIEMs, thus far, the evaluation of PIEM characteristics and the validation of RED performance have only been conducted for the small RED stack. 

In the present work, we fabricated various compositions of large IEMs for the variation of resistance and permselectivity of PIEMs to analyze the relationship between PIEM properties and large-scale RED stack performance. In addition, to evaluate the relationship between the characteristics of PIEMs and stack size, differing sizes of RED stacks (5 × 5 cm^2^ and 15 × 15 cm^2^, total membrane area 0.025 m^2^ and 0.225 m^2^, respectively) were prepared. The performance of the RED stack was evaluated by measuring the electrical power on the single RED stack according to the membrane composition and stack size under the same linear velocity. Then, we analyzed the correlation factors of electrical power, electrical resistance, and electrical current. In particular, the effects of stack size and PIEM composition on the internal RED stack resistance are discussed in detail. Based on these results, we determined that the electrical resistance of PIEMs is a more dominant factor than its permselectivity for the production of high electrical power from the large-scale RED stack for commercialization.

## 2. Materials and Methods

### 2.1. Reverse Electrodialysis (RED) System

**Figure 1:**
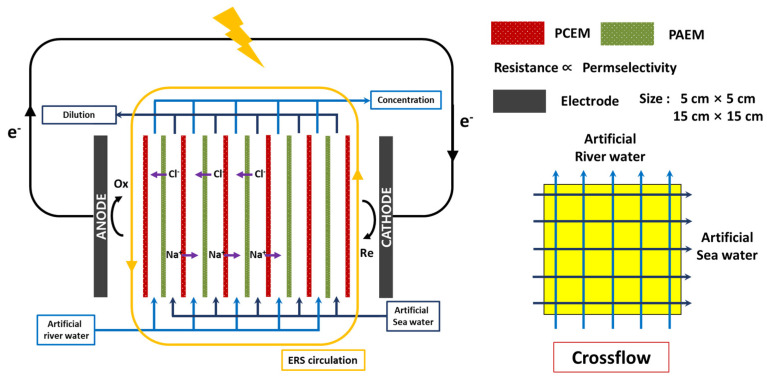
Schematic illustration of the reverse electrodialysis (RED) process according to the variation in properties of the ion exchange membrane as well as electrode size. ERS: electrode rinse solution.

To evaluate RED performance according to the size of the RED stack with various PIEM compositions, different sizes of two cross-flow-type RED stacks (small size: 5 × 5 cm^2^; large size: 15 × 15 cm^2^) were prepared (LAPINION, Seoul, South Korea; Figure 1). The endplates of the RED stacks were fabricated using polyvinylchloride (PVC), and each inlet and outlet port was installed on the endplates. The RED stacks consisted of five unit cells, and each unit cell was composed of a pore-filling cation exchange membrane (PCEM), pore-filling anion exchange membrane (PAEM), mesh-type spacers (open area: 81.3%, thickness: 100 μm, DS Mesh, Seoul, South Korea), and polytetrafluorethylene (PTFE) gaskets (thickness: 100 μm, Tommy Hecco, Seoul, South Korea). The PCEMs and PAEMs were fabricated using the methods described in previous works [24,25]; the detailed fabrication method is explained in the following section. The 4:1, 8:1, and 12:1 compositions of the PCEM and PAEM mean weight ratio between the electrolyte monomer and crosslinking agent were used to prepare the PCEM and PAEM, as shown in Table 1. The RED stacks with PIEMs of 4:1, 8:1, and 12:1 compositions were assembled with PCEMs and PAEMs with a 4:1 composition, PCEMs and PAEMs with a 8:1 composition, and PCEMs and PAEMs with a 12:1 composition. To inhibit the transport of the electrode rinse solution (ERS) into the feed solution channels for each RED stack, PCEMs were used as shielding membranes at both sides of the cathode and anode. A Pt-coated Ti mesh (Wesco electrode, thickness: 1 mm, coating layer: <3 µm, South Korea) was used for the electrodes, while an aqueous mixed solution of 0.05 M K_4_Fe(CN)_6_ and 0.05 M K_3_Fe(CN)_6_ (EP grade, Daejung, South Korea) was used as the ERS for the reversible electrochemical reaction without consumption of the redox species. The ERS was recirculated between electrode compartments in the RED stacks using the same peristaltic pump. The flow rates of the ERS in the small and large RED stack were 50 mL/min and 150 mL/min, respectively. 

Artificial river water and seawater water were simulated using 0.017 M NaCl and 0.5 M NaCl, respectively. In the present study, a cross-flow type RED stack was used as shown in Figure 1. In general, this type of RED stack presents higher efficiency than the co-current type stack; however, it has a low membrane power density. Based on the modeling results, the counter-flow RED stack is much efficient than the cross-flow RED stack; however the cross-flow RED stack is much more practical than the cross-flow RED stack [35]. The artificial feed solutions were pumped into the RED stack using two peristaltic pumps (Masterflex L/S Digital Drive, Cole-Parmer, Vernon Hills, IL, USA). The flow rates of the feed solutions in the small (5 × 5 cm^2^) and large (15 × 15 cm^2^) RED stacks were 30 mL/min and 90 mL/min, respectively. Under these conditions, each small and large RED stack presented the same linear velocity of 2.0 cm/s. In general, a laboratory-scale RED stack has the highest net power density near a linear velocity of 1.0 cm/s [36]. However, with a higher linear velocity, the performance of the IEM for the RED stack (i.e., membrane power density) is improved because of the more stable salinity gradients across the IEM. Based on the experimental validation, we set the linear velocity as a constant (i.e., 2.0 cm/s) to analyze the effect of IEM characteristics and stack size. The operation and performance evaluation of the RED system and RED stack assembled with the PIEMs, respectively, were conducted at room temperature.

### 2.2. Evaluation of the RED Stack Performance

The electrical power of each RED stack was measured by linear sweep voltammetry (LSV) with a sweep rate of 40 mV/s set using a potentiostat (ZIVE SP5, Wonatech, South Korea). For the sake of simplicity, gross power (P) on the single RED stack was calculated by Ohm’s law (Equation (1)). During the estimation of gross power, pumping power loss by pressure drop is not considered. In general, the highest gross power on the RED stack could be achieved when the electrical potential was equal to half of the open circuit voltage (OCV). In addition, under the same conditions, the internal electrical resistance of the RED stack was equal to the external electrical resistance of the RED stack (Equation (1)). The power density was calculated by normalizing the electrical power for the total membrane area (Equation (2)):

(1) P=V×I=V2Ru=(VO)24Ru

(2) Pd=PAm

where P is the gross power (W), V is the electrical potential (V), I is the electrical current (A), V_O_ is the OCV (V), Ru is the electrical resistance of the external load (Ω), P_d_ is the power density (W/m^2^), and A_m_ is the total membrane area in the RED stack (m^2^).

The electrochemical membrane potential in the RED stack under the ideal condition can be calculated using the Nernst equation (Equation (3)) [37,38,39,40]:

(3) Em=(αavg)RTzF[lnAHCALC]

where Em is the membrane potential by salinity gradients (V), α_avg_ is the average permselectivity of the CEM and AEM, R is the gas constant (8.314 g/mol-K), T is the absolute temperature (K), z indicates the valence of the ions (assumed to be 1 in a 1:1 binary solution of NaCl), F is the Faraday constant (96,485 C/mol), and A_HC_ and A_LC_ represent the activities of the feed solutions.

The internal resistance of the RED stack (R_i_) consists of the ohmic resistance (R_ohmic_), non-ohmic resistance (R_non-ohmic_) such as the boundary layer resistance (R_BL_), and bulk layer resistance based on concentration variations (R_Δc_) (Equation (4)). The contributions of ohmic and non-ohmic resistance to the internal resistance of small and large RED stacks with respect to PIEM characteristics were calculated using the following equations (Equations (5)–(7)) [34]:

(4) $$R_{i}=R_{\mathrm{ohmic}}+R_{\mathrm{non}\text{-}\mathrm{ohmic}}=R_{\mathrm{ohmic}}+R_{\Delta c}+R_{\mathrm{BL}}$$

(5) Rohmic=NA(RAEM+RCEM+dHCκHC+dLCκLC)

(6) RΔc=(αavg·R·TF·J)ln(ΔaLCΔaHC)

(7) $$\Delta a_{\mathrm{HC}}=1-\left(\frac{J\cdot L}{F\cdot q_{\mathrm{HC}}\cdot c_{\mathrm{HC}}}\right),\;\Delta a_{\mathrm{LC}}=1+\left(\frac{J\cdot L}{F\cdot q_{\mathrm{LC}}\cdot c_{\mathrm{LC}}}\right)$$

where N is the number of unit-cell pairs; A is the membrane projection area (cm^2^); R_AEM_ and R_CEM_ are the area resistances of the AEM and CEM, respectively (Ω-cm^2^); d_HC_ and d_LC_ are the thicknesses of the high-concentrated (HC) and low-concentrated (LC) flow channels, respectively (cm); κ_HC_ and κ_LC_ are the molar conductivities of the HC and LC feed solutions, respectively; J is the current density (mA/cm^2^); L is the cell length (cm); q is the flow rate (cm^3^/s); and c is the concentration of the feed solution (mmol/cm^3^).

For simplicity, the ohmic resistance of the RED stack can be estimated by linear integration of the ohmic resistance of each component in the unit cell, such as flow channels and IEMs. The LC flow channel significantly contributes to the ohmic resistance of the RED stack (Equation (5)). The bulk layer resistance (R_Δc_), which originated from the concentration change of the feed solutions along the flow channels, can be calculated using Equation (6). Although the direct calculation of the intensity of the boundary layer resistance (R_BL_) is quite complicated, the intensity can be derived from Equation (4) by substituting the known values of ohmic resistance and bulk layer resistance. The non-ohmic resistance is affected by the current density (J) of the RED stack. Thus, it can be changed dramatically by varying the stack composition and electrical current of the RED stack.

### 2.3. Fabrication and Characterization of Pore-Filling Ion Exchange Membranes (PIEMs)

The PCEMs and PAEMs used in the study were also fabricated based on previously reported methods [24,25]. We designed PIEMs such that the permselectivity was proportional to the resistance. The permselectivity of all PIEMs was designed to be higher than the minimum permselectivity of commercial IEMs (>90%). The compound (3-acrylamidopropyl) trimethylammonium chloride (75 wt% in water, ATAC, KJ chemical Co., Tokyo, Japan) and a mixture of 2-acrylamido-2-methyl-1-propanesulfonic acid (AMPS, Toagosei, Japan) and 2-acrylamido-2-methyl-1-propanesulfonic acid sodium salt (solution with 50 wt% in H_2_O, AMPS–Na, Toagosei, Tokyo, Japan) were used as electrolytes for preparing the PAEMs and PCEMs, respectively. Piperazine diacrylamide (PDA), used as a crosslinking agent, was purchased from Richest Group (Shanghai, China). The photoinitiator for the photo-radical polymerization of acrylamide groups using the electrolyte and crosslinking agent was 2-hydroxy-2-methylpropiophenone (10 wt% in MeOH, TCI Chemicals, Tokyo, Japan). The dry weight ratios between the electrolyte and crosslinking agent for preparing the PCEMs and PAEMs with various performances are listed in Table 1. The photoinitiator was added with a 144:0.1 weight ratio of the electrolyte and photoinitiator. The crosslinking agent was added to the electrolyte solutions according to the composition in Table 1. The electrolyte and crosslinking agent mixtures were stirred for 2 h to obtain clear and homogeneous solutions; then, the photoinitiator was added to each solution. 

A porous polyethylene (PE, pore volume: 43%, pore size: 60 nm, thickness: 16 µm, W-Scope, Tokyo, Japan) substrate pretreated with a clear solution of Tergitol 15-S-7 (Dow Chemical, Midland, MI, USA) and deionized water at a 1:100 weight ratio was prepared as a supporter for the fabricated PCEMs and PAEMs. For pretreatment, PE substrates were soaked in the solution for 1 min. Subsequently, wet PE substrates were dried using an air gun set at a temperature of 50 °C. The PE substrates hydrophilized by pretreatment were cut to dimensions of 30 × 30 cm^2^. The substrate was then impregnated with mixed solutions of the electrolyte, crosslinking agent, and photoinitiator. The PE substrates wetted by the solutions were sandwiched between two polyethylene terephthalate (PET) films to block contact between the solution and atmospheric oxygen. For photo-radical polymerization of acrylamide groups of electrolytes and crosslinking agents in PE substrates, the above assemblies were irradiated with an ultraviolet (UV) light (metal-halide lamp, main wavelength: 365 nm) for 3 min. After photopolymerization, the top and bottom PET films from the assemblies were removed. The obtained PCEMs and PAEMs were then polished to remove the polymerized electrolytes and crosslinking agent from the PIEM surface. In addition, to eliminate impurities, the PIEMs were thoroughly washed with deionized water. The thickness of all PIEMs measured with a micrometer (Mitutoyo, Kawasaki, Japan) was approximately 16–17 µm regardless of their composition. The ion exchange capacity (IEC), permselectivity, and resistance of the fabricated PIEMs were measured using previous experimental methods [24,25]. The detailed experimental methods are summarized in the Supporting Material (Figure S1).

## 3. Results and Discussion

### 3.1. Effects of Composition of PIEMs on RED Performance

As shown in Table 1, the IECs of the PCEMs and PAEMs decreased with the decreasing hydrophilic electrolyte and increasing hydrophobic crosslinking agent with respect to their compositions. Furthermore, similar variations of permselectivity and resistances of the PCEMs and PAEMs were shown within the same composition variations of PCEMs and PAEMs. This variation in the permselectivity and resistances of the PCEMs and PAEMs is likely related to the crosslinking density and electrolyte content in the PIEMs. The cross-linking density increased upon increasing the crosslinking agent and decreasing the electrolyte content in the PIEMs. The increment of the cross-linking density led to an increase in permselectivity and resistance of the PIEMs [32]. In addition, the fabricated PIEMs showed lower resistance compared with the commercialized IEMs regardless of their compositions [25]. The low resistances of the PIEMs are attributed to their low thickness (16–17 μm) as compared with those of commercialized IEMs (Fujifilm Type-1 CEM 135 μm, Fujifilm Type-1 AEM 125 μm). The PAEM with similar compositions had excellent chemical stabilities upon immersion in the aqueous 5 M NaOH solution at 50 °C for 1500 h as a harsh condition compared with RED operation. The chemical stability of the PCEM was verified under fuel cell operation with a harsher condition than RED operation. In addition, as previously reported, the PIEM had a high tensile strength of over 100 MPa originated from the high tensile strength of porous polyethylene [41,42].

**Figure 2:**
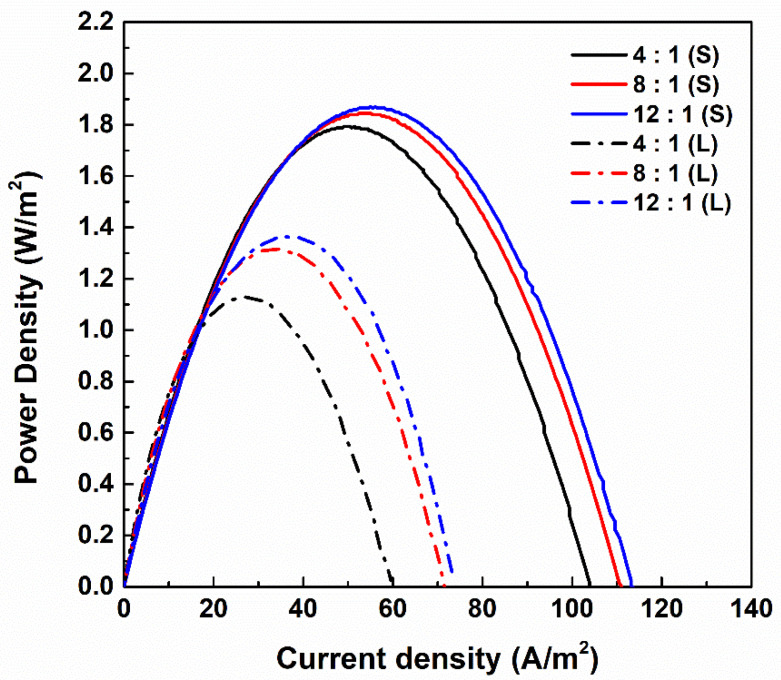
Gross power density of large and small RED stacks according to the composition of pore-filling ion exchange membranes (PIEM). “S” and “L” indicate the results obtained from small and large RED stacks, respectively.

Figure 2 shows the gross power density of large and small RED stacks assembled with PIEMs of various compositions. The maximum power densities of the small RED stack, which consisted of PIEMs with 4:1, 8:1, and 12:1 compositions, were 1.794 W/m^2^, 1.845 W/m^2^, and 1.870 W/m^2^, respectively. The maximum power density of the small RED stack assembled with Fujifilm Type-1 CEM/AEM was previously reported as ~1.4 W/m^2^. The higher power density of the PIEMs was attributed by their lower resistance and similar permselectivity compared with those of Fujifilm Type-1 CEM/AEM [25,32]. When compared with another commercial IEM, the PIEMs showed higher power density [13]. The large RED stack assembled with PIEMs of the same compositions were 1.129 W/m^2^, 1.316 W/m^2^, and 1.364 W/m^2^. Furthermore, the current densities of the small RED stack were higher than those of the large stack, and the current densities of the stack increased with increasing electrolyte concentration in the PIEMs (12:1 > 8:1 > 4:1), regardless of the stack size. As mentioned earlier, increments of RED stack size with the same linear velocity lead to a reduction in salinity gradients across the IEMs and decreases in the power densities and current densities of the large RED stack. It is worth noting that the increment ratio of the electrolyte in the PIEMs was positive for the power and current density of the RED stack regardless of the stack size. The positive effects of lower electrical resistance of IEMs on the RED stack performance have already been reported by previous researchers [5,13,24,31,32,33]. However, to the best of our knowledge, this is the first approach to validating the effects of the electrolyte ratio in a membrane matrix on RED performance. Increasing the ratio of the electrolyte from 4:1 to 12:1 at PCEM reduced the permselectivity by 4.2% and electrical resistance by 43.6%. In addition, increasing the ratio of the electrolyte from 4:1 to 12:1 at PAEM reduced the permselectivity by 7.14% and electrical resistance by 48.0%. The electrical resistance that was remarkably reduced compared with permselectivity improved the electrical power on the RED stack. To commercialize the RED stack as a power generation device, it is necessary to conduct research on the correlation between the properties of the IEMs and the power generation performance of the large RED stack.

### 3.2. Correlation between Compositions of Ion Exchange Membranes (IEMs) and RED Performance

**Figure 3:**
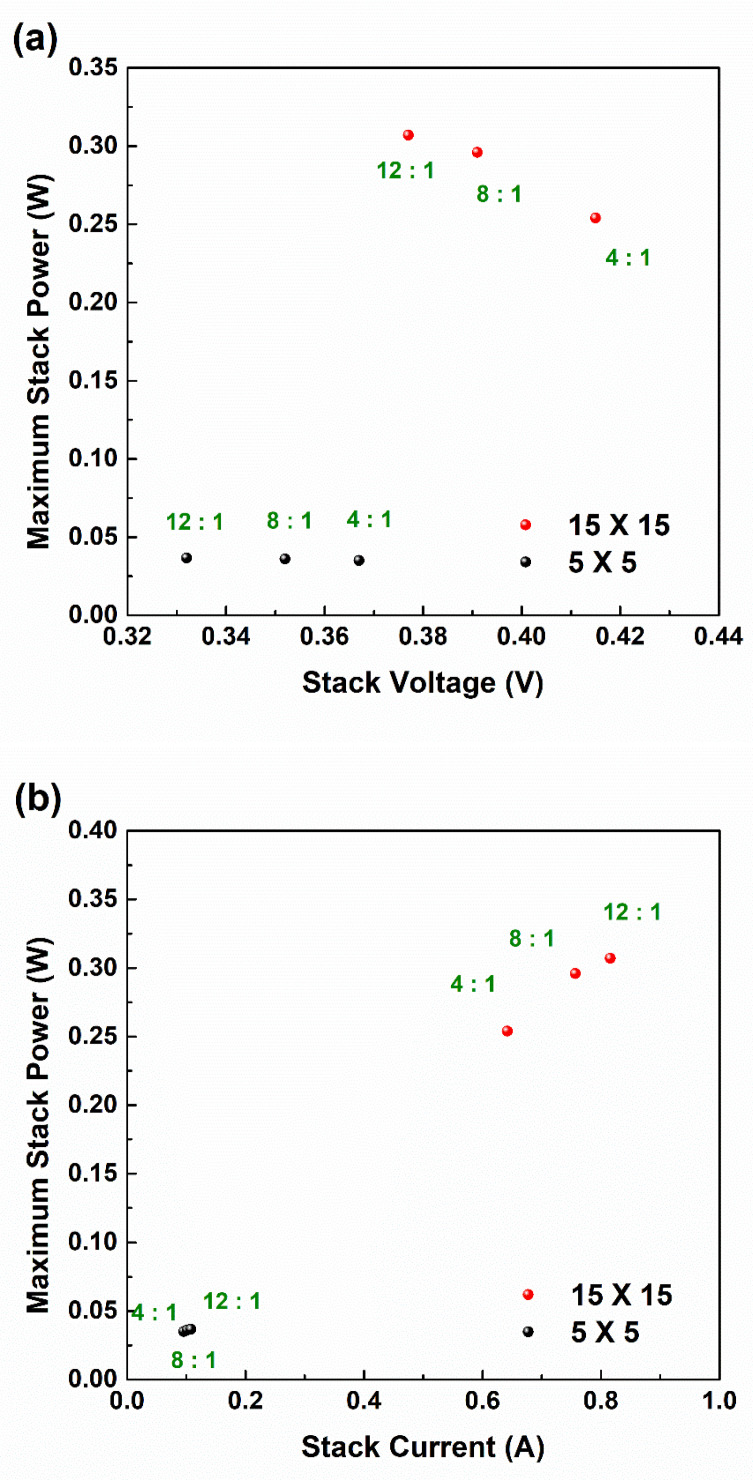
Relationship between maximum power and (**a**) stack voltage, and (**b**) stack current, according to PIEM composition and RED stack size.

Figure 3a,b show the voltage–power (V–P) and current–power (I–P) plots according to the compositions of the IEMs of the small and large RED stacks, respectively. The results of the highest power on the RED stack according to experimental conditions were adopted from general current–voltage (I–V) curves (Figure S1). As shown in Figure 3a, the maximum powers from the small RED stack according to the 4:1, 8:1, and 12:1 PIEM compositions were 0.0351 W, 0.0361 W, and 0.0367 W, respectively. In addition, the large RED stack with PIEMs of the same compositions showed maximum powers of 0.254 W, 0.296 W, and 0.307 W, respectively. In the present study, a large RED stack had a nine times larger active area of the electrode than a small RED stack; thus, power generation on the large RED stack would be nine times higher than on the small RED stack, assuming a linearity between stack size and power generation. However, there were certain differences between the experimental results and the expected value on the large RED stack. The power loss ratios by enlarging stack size were 19.6%, 8.9%, and 7.1% at RED stacks with 4:1, 8:1, and 12:1 PIEM compositions. The reduction in power losses according to the IEM composition on the large RED stack is primarily attributed to the parasitic ionic current across the IEMs. The salinity gradient across the IEMs is the driving force of ion transport, and it is constant regardless of the IEM composition. However, the IEC value determines the total amount of the escapable ions in the high concentration compartment. The ions excluded from the ion exchange are still affected by salinity gradient, thus generating the parasitic ionic current. 

As represented in the stack V–P plot of Figure 3a (also refer to Figure S1), the differences of electrical voltage according to composition of the PIEM were not significant to the electrical power on the RED stack. This phenomenon is much clearer for the small RED stack, where the parasitic ionic current was not dominant. In addition, increasing the stack size did not lead to a significant electrical potential difference in the RED stack (i.e., electrical voltage) between the small and large RED stacks (less than 0.05 V). This indicates the optimal permselectivity to achieve maximum electrical power on the RED stack with less significant correlation between power generation and electrical voltage according to the stack size.

As shown in the stack I–P plot of Figure 3b (also refer to Figure S1), the change in the electrical current and power according to the PIEM composition ratio was smaller in the small RED stack than in the large RED stack. In the large RED stack, the resistances of the PIEMs partially contributed to the electrical current and power. In particular, the decrease in the resistance of the PIEMs in large RED stacks led to a sharp increase in the electrical current; thus, the power of the RED stack clearly increased. This phenomenon could also be described by adopting faradaic ionic current, as already mentioned earlier.

**Figure 4:**
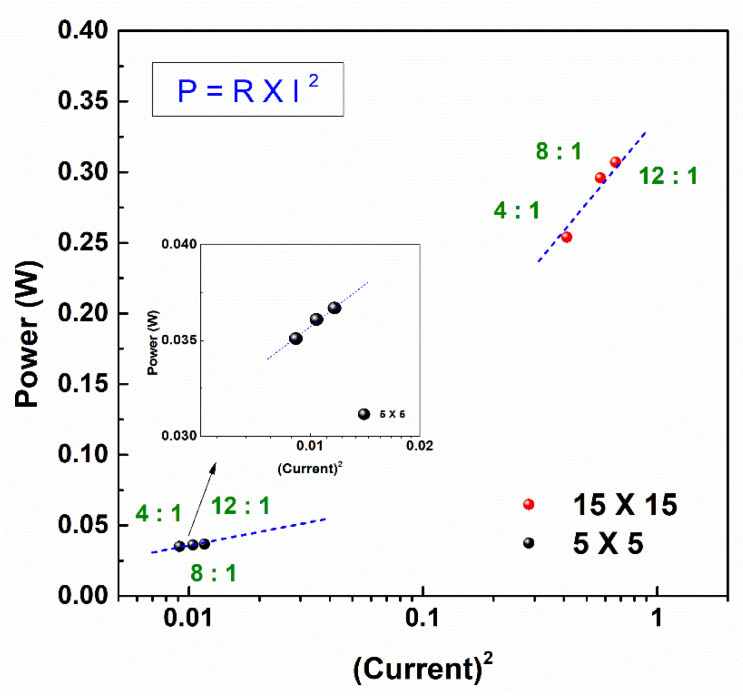
Relationship between the square of stack current and power according to PIEM composition and RED stack size.

Figure 4 shows the correlation between the square of electrical current (I2) and electrical power (P) according to the PIEM composition and RED stack size, supporting the results shown in Figure 3. In Figure 4, each slope of the plots indicates the electrical resistance of the RED stacks, including ohmic and non-ohmic resistances. In a small RED stack, variations in electrical resistances of RED stacks according to IEM compositions were smaller than in a large RED stack. This could not be explained by Ohm’s law because enlarging the active area of IEM allows for increased ion transport. Therefore, the hindered electrical resistance impedes ion transport regardless of the ohmic resistance of the IEMs. In general, this is called the non-ohmic resistance. In this specific case, non-ohmic resistance had a more significant effect on ion transport than the ohmic resistance of the IEMs. Variations in the electrical resistances of PIEMs by composition led to a sharp change in the large RED stack resistance. The abrupt variations in the stack resistance according to the membrane composition in the large RED stack were closely related to the higher sensitivity of the electrical current rather than the electrical potential of large stacks, as represented in Figure 3.

### 3.3. Current–Voltage (I–V) Curves According to RED Stack Size

**Figure 5:**
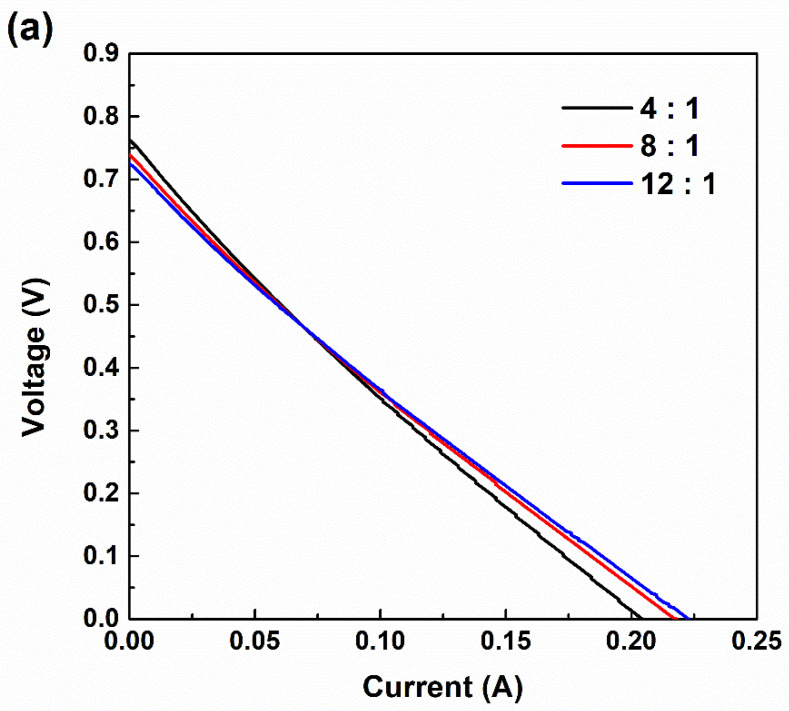

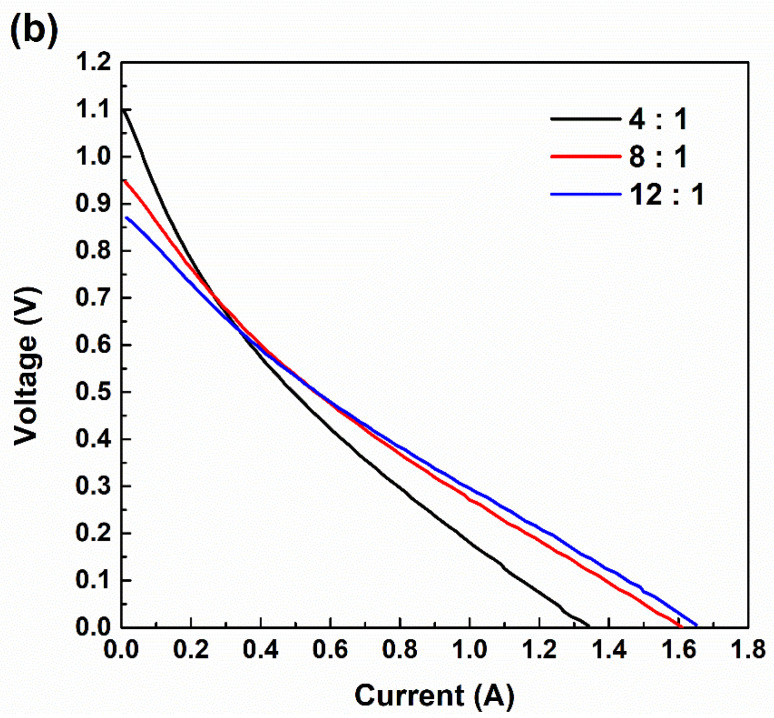
Current–voltage (I–V) curves of RED stacks with sizes (**a**) 5 × 5 cm^2^ and (**b**) 15 × 15 cm^2^, according to PIEM compositions.

As shown in Figure 5, the I–V curves were plotted according to the RED stack size and PIEM compositions for a detailed analysis of the ohmic and non-ohmic resistances of the RED stack. Regardless of the RED stack size, the OCV values, which correspond to the y-intercepts of the I–V curves, increased according to the permselectivity increments of the PIEMs. According to the Nernst equation, increasing the RED stack size should cause the OCV to decrease due to the concentration difference decrease between feed waters. However, in Figure 5, a higher OCV is observed in the large RED stack, as compared with the small RED stack. In order to explain this contrasting phenomenon, additional research is required on the basis of further experiments. The slopes of the I–V curves were reduced by decreasing the electrical resistance of the PIEMs, and the maximum electrical currents of the RED stack increased according to the electrical resistance reduction of the PIEMs. These phenomena were prominent in the I–V curves of the large RED stack (Figure 5b). Remarkably, the decisive difference between the I–V curves evaluated for the large and small RED stacks was determined to be the linearity. The small RED stack maintained the linearity of curves independent of the composition of the PIEMs, indicating that the small stack ideally followed ohmic behavior, such that the maximum power of the small stack could be obtained at the point of half the electrical potential of the OCV. A considerably small ohmic resistance (< 0.8 Ω-cm^2^) of the PIEMs led to ohmic behavior of the small RED stack. It also revealed that there was no significant non-ohmic energy loss in the membrane–solution interface in the small RED stack, such as owing to concentration polarization. On the other hand, the I–V curves of the large RED stack presented less linearity than the small RED stack. This non-linearity of the I–V curves indicates that the contribution of non-ohmic resistance to the total stack resistance increased by enlarging the RED stack size. In particular, the non-linearity of the I–V curves showed an increasing tendency with respect to permselectivity increments and decrease in the electrical resistance of PIEMs (12:1 → 8:1 → 4:1 compositions). This tendency indicates that the contribution of non-ohmic resistance to the total stack resistance of the large RED stack assembled with the 4:1 PIEM composition was the greatest among the RED stacks.

### 3.4. Ohmic and Non-Ohmic Resistances According to RED Stack Size

**Figure 6:**
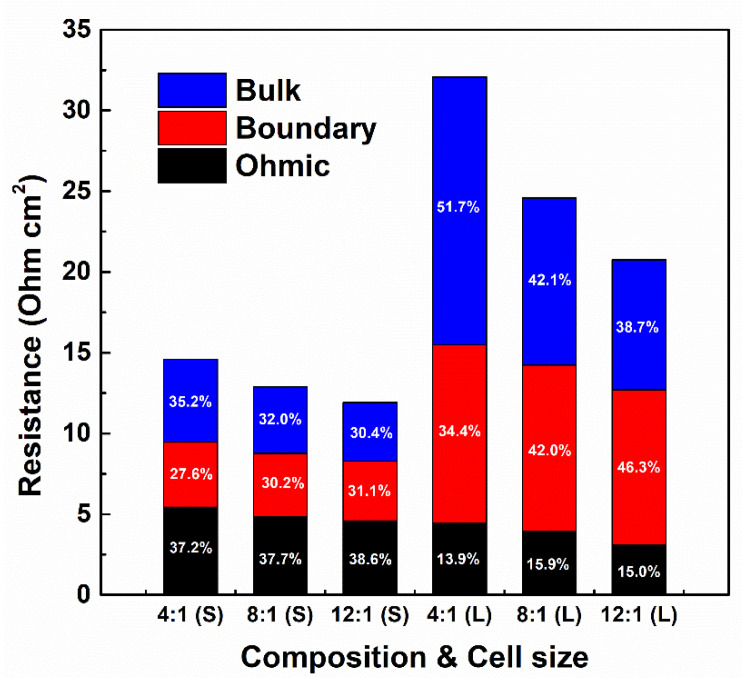
Internal resistance contribution with respect to PIEM composition and RED stack size.

Figure 6 represents the contributions of internal resistance components with respect to the stack size and PIEM compositions. By enlarging the RED stack size, the internal resistances were increased; thus, the contributions of each component to the internal resistances of the RED stacks were dramatically changed. Assuming that the permselectivity and electrical resistance of the IEMs are constant regardless of the concentration gradient across the IEM, the change in internal resistance and its composition ratio are determined to be mainly related to the ion transport considering the experimental conditions. The contributions of the ohmic resistance to the internal resistance of the RED stack are determined by the properties of the materials (e.g., electrical conductivity of IEMs, electrical conductivity of feed solutions, etc.) and configurations of the RED stack (e.g., thickness of membranes, inter-membrane distances, etc.) [13,34]. By enlarging the stack size, the ohmic resistance was slightly decreased because the active site area of the PIEMs increased and the total number of ions transported through the PIEMs also increased. According to Ohm’s law, the ohmic resistance, which enlarges the active area of the conductive material, also reduces its electrical resistance. Furthermore, at the same linear velocity, increments in stack size allowed for enhanced ion transport through the IEMs and increased the concentration of the LC effluent. With the lower electrical resistance of IEMs, ion transport was determined to be far more prominent. Consequently, the electrical conductivity in the LC flow channel, which significantly contributes to the ohmic resistance of the RED stack, was increased.

Unlike the decreasing tendency of ohmic resistances by enlarging the stack size, the boundary layer resistances of the large RED stack were more dramatically increased than those of the small RED stack, regardless of the PIEM compositions. The increase in the boundary layer resistance is most notably attributed to the stagnation of ion transfer on the membrane–electrolyte interface. According to Nernst–Planck analysis, ions are mainly transferred across the PIEMs in the RED stack via diffusion, and decreasing the diffusion rate by lowering salinity gradients might increase the boundary layer resistance [43]. In this study, the reduction in salinity gradients across the PIEMs decreased the ion transfer rate and was prominent in the large RED stack. The boundary layer resistance was decreased from 4.02 Ω-cm^2^ to 3.70 Ω-cm^2^ (4:1 to 12:1) in the small RED stack and from 11.04 Ω-cm^2^ to 9.61 Ω-cm^2^ (4:1 to 12:1) in the large RED stack. However, the contribution of the boundary layer resistance to the internal resistance of the RED stack was distinctly increased, from 27.6% (4:1) to 31.1% (12:1) in the small RED stack and from 34.4% (4:1) to 46.3% (12:1) in the large RED stack. Moreover, the decrease in the boundary layer resistance by increasing the composition ratio of PIEMs is acceptable because ion transport is faster in PIEMs with a high composition ratio. As the size of the RED stack increases, these effects are intensified due to the decrease in the salinity gradients between the HC and LC feed solutions, resulting in a larger change in the boundary layer resistance due to the resistance reduction of the PIEMs. 

The bulk layer resistance is the variation of concentration in the flow channel, which is caused by ion transport from the HC flow channel to the LC flow channel through the active surface of the IEMs. As is already known, the LC flow channel has the highest electrical resistance in the RED stack [13,34,44]. The intensity of the bulk layer resistance is determined by the ion transport rate and retention time of the feed solutions. During RED stack operation, the ion transport rate could be estimated based on the electrical current by assuming that electro-neutrality and charge neutrality were satisfied across the PIEMs and electrodes. In addition, the ion transport rate and retention time are significantly related to the compositions and active areas of the IEMs, respectively. In this study, the bulk layer resistances were sensitive to changes in the stack size and PIEM compositions; these results were noticeable in large RED stacks. In particular, the contribution of the bulk layer resistance to the internal resistance of the RED stack was 51.7% in the large RED stack with a 4:1 PIEM composition. The entirety of the bulk layer resistance increment in the large RED stack was attributed to slow ion transport from the HC to LC feed solutions through the PIEMs, which is attributed to reductions in the salinity gradients between feed solutions by enlarging the stack size. However, the bulk layer resistance in the large RED stack was dramatically reduced with a decrease in the electrical resistance of the PIEMs. This phenomenon indicates that, even with decreasing salinity gradient between the feed solutions in the large RED stack, the low electrical resistance of the PIEMs could still have a significant effect on the rapid transport of ions through the PIEMs in the large RED stack, as compared with the small stack.

### 3.5. Conductivities of Influents and Effluents from the RED Stacks

**Figure 7:**
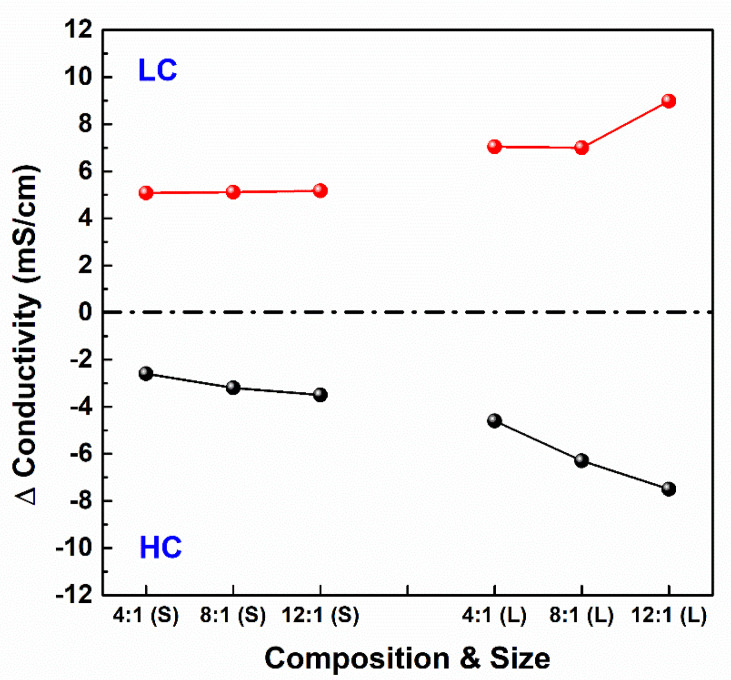
Conductivity difference between the inlet and the outlet of both low-concentrated (LC) feed water and high-concentrated (HC) feed water according to the PIEM composition and RED stack size.

As mentioned earlier, the ion transport rate is affected by PIEM composition and stack size. Consequently, the rate determines the contributions of the internal resistance of the RED stack. Figure 7 shows the electrical conductivity differences between the influents and effluents according to the PIEM composition and stack size. The major parameters for determining the electrical conductivity of the effluent are the electrical resistance of the IEMs and the retention time in the RED stack. A high composition ratio of the IEM allowed for rapid ion transport and increased the electrical conductivity of the effluents. In addition, enlarging the stack size increased retention time as well as the electrical conductivity of the effluents. Additionally, the asymmetricity between HC and LC may be attributed to the electro-osmosis or osmosis of the water flux. Furthermore, asymmetric ion transport may have occurred to impedance on the IEMs or charge neutrality. Although it is not a significant problem in the present study, it should be considered in the pilot-scale RED stack, which uses natural seawater containing multiple ionic species. The variation tendency of the electrical conductivities was similar to that of the internal resistance of the RED stack (Figure 6), such that the low electrical resistance of the PIEMs in the large RED stack enhanced the ion transport rate through the PIEMs as compared with the transport rate of the small stack. Considering the results from Figure 6 and Figure 7, the compositions and electrical resistance (i.e., ion transport rate) of PIEMs have significant effects on the performance of the RED stack and should be considered as key factors for upscaling the RED stack and its commercialization.

## 4. Conclusions

In the present study, various compositions of PIEMs were prepared and adopted for small and large RED stacks. The performance of the RED stacks was evaluated in terms of the maximum power density according to the stack size. To determine the correlation between the compositions of PIEMs and stack size, the contributions of both ohmic and non-ohmic resistances were analyzed. The major findings of this study are as follows:

- Increasing the electrolyte content in the composition of PIEMs reduced the electrical resistance and significantly affected the power density of the RED stack. The maximum power densities of 1.870 W/m^2^ and 1.364 W/m^2^ were achieved for the small and large RED stacks with a 12:1 PIEM composition, respectively.
- Enlarging the stack size significantly affected the power generation with a significant contribution of non-ohmic resistance to the internal resistance of the RED stack.
- The PIEM composition significantly affected the non-ohmic resistance of the RED stack. In the large RED stack, the bulk layer resistance contributed to 51.7% of the internal resistance of the RED stack with a 4:1 PIEM composition.
- The variations in non-ohmic resistances were attributed to the ion transport rate across the PIEMs with a salinity gradient reduction by enlarging stack size and ion transport enhancement by lowering electrical resistance according to PIEMs as major factors.
- Permselectivity was less sensitive to RED performance than electrical resistance when exceeding 90%.

## Figures and Tables

**Table 1:** Characteristics of pore-filling cation exchange membranes (PCEMs) and pore-filling anion exchange membranes (PAEMs) according to the composition of the electrolyte and crosslinking agent.

Notation		Composition (Weight Ratio)			Thickness (μm)	IEC (meq/g)	Permselectivity (%)	Resistance (Ω·cm^2^)
		AMPS–Na + AMPS	ATAC	PDA				
Fujifilm Type-1 CEM [25]		-	-	-	135	1.83	97.4	2.10
PCEM	4:1	4	-	1	16	1.54	99.9	0.75
	8:1	8	-	1		1.67	98.7	0.49
	12:1	12	-	1		1.85	93.7	0.37
Fujifilm Type-1 AEM [25]		-	-	-	125	1.84	93.8	1.22
PAEM	4:1	-	4	1	16	1.50	99.5	0.77
	8:1	-	8	1		1.64	97.1	0.48
	12:1	-	12	1		1.81	93.4	0.37

## Data Availability

Not applicable.

## Supplementary Materials

The following are available online at https://www.mdpi.com/article/10.3390/membranes11080609/s1, Figure S1: (a) Stack current–power curves, and (b) stack voltage–power curves according to a composition of PIEMs and size of RED stacks. “S” and “L” in the figure indicate the results obtained from the small and large RED stacks, respectively.

## References

[1] Logan B.E., Elimelech M. (2012). Membrane-based processes for sustainable power generation using water. Nature.

[2] Yip N.Y., Brogioli D., Hamelers H.V., Nijmeijer K. (2016). Salinity Gradients for Sustainable Energy: Primer, Progress, and Prospects. Environ. Sci. Technol..

[3] Jia Z., Wang B., Song S., Fan Y. (2014). Blue energy: Current technologies for sustainable power generation from water salinity gradient. Renew. Sustain. Energy Rev..

[4] Mei Y., Tang C.Y. (2018). Recent developments and future perspectives of reverse electrodialysis technology: A review. Desalination.

[5] Hong J.G., Zhang B., Glabman S., Uzal N., Dou X., Zhang H., Wei X., Chen Y. (2015). Potential ion exchange membranes and system performance in reverse electrodialysis for power generation: A review. J. Membr. Sci..

[6] Ortiz-Imedio R., Gomez-Coma L., Fallanza M., Ortiz A., Ibañez R., Ortiz I. (2019). Comparative performance of Salinity Gradient Power-Reverse Electrodialysis under different operating conditions. Desalination.

[7] Vermaas D.A., Kunteng D., Veerman J., Saakes M., Nijmeijer K. (2014). Periodic feedwater reversal and air sparging as antifouling strategies in reverse electrodialysis. Environ. Sci. Technol..

[8] Nam J.Y., Hwang K.S., Kim H.C., Jeong H., Kim H., Jwa E., Yang S., Choi J., Kim C.S., Han J.H. (2019). Assessing the behavior of the feed-water constituents of a pilot-scale 1000-cell-pair reverse electrodialysis with seawater and municipal wastewater effluent. Water Res..

[9] Post J.W., Veerman J., Hamelers H.V.M., Euverink G.J.W., Metz S.J., Nymeijer K., Buisman C.J.N. (2007). Salinity-gradient power: Evaluation of pressure-retardedosmosis and reverse electrodialysis. J. Membr. Sci..

[10] Jwa E., Yun Y.-M., Kim H., Jeong N., Hwang K.S., Yang S.C., Nam J.-Y. (2020). Energy-efficient seawater softening and power generation using a microbial electrolysis cell-reverse electrodialysis hybrid system. Chem. Eng. J..

[11] Zhang H., Sun C. (2021). Cost-effective iron-based aqueous redox flow batteries for large-scale energy storage application: A review. J. Power Sources.

[12] Jang J., Kang Y., Han J.-H., Jang K., Kim C.-M., Kim I.S. (2020). Developments and future prospects of reverse electrodialysis for salinity gradient power generation: Influence of ion exchange membranes and electrodes. Desalination.

[13] Kim H.-K., Lee M.-S., Lee S.-Y., Choi Y.-W., Jeong N.-J., Kim C.-S. (2015). High power density of reverse electrodialysis with pore-filling ion exchange membranes and a high-open-area spacer. J. Mater. Chem. A.

[14] Geise G.M., Hickner M.A., Logan B.E. (2013). Ionic resistance and permselectivity tradeoffs in anion exchange membranes. ACS Appl. Mater. Interfaces.

[15] Fan H., Yip N.Y. (2019). Elucidating conductivity-permselectivity tradeoffs in electrodialysis and reverse electrodialysis by structure-property analysis of ion-exchange membranes. J. Membr. Sci..

[16] Merino-Garcia I., Kotoka F., Portugal C.A., Crespo J.G., Velizarov S. (2020). Characterization of poly (Acrylic) acid-modified heterogenous anion exchange membranes with improved monovalent permselectivity for RED. Membranes.

[17] Guler E., Zhang Y., Saakes M., Nijmeijer K. (2012). Tailor-made anion-exchange membranes for salinity gradient power generation using reverse electrodialysis. ChemSusChem.

[18] Hong J.G., Chen Y. (2014). Nanocomposite reverse electrodialysis (RED) ion-exchange membranes for salinity gradient power generation. J. Membr. Sci..

[19] Tufa R.A., Piallat T., Hnát J., Fontananova E., Paidar M., Chanda D., Curcio E., di Profio G., Bouzek K. (2020). Salinity gradient power reverse electrodialysis: Cation exchange membrane design based on polypyrrole-chitosan composites for enhanced monovalent selectivity. Chem. Eng. J..

[20] Güler E., Elizen R., Vermaas D.A., Saakes M., Nijmeijer K. (2013). Performance-determining membrane properties in reverse electrodialysis. J. Membr. Sci..

[21] Güler E., van Baak W., Saakes M., Nijmeijer K. (2014). Monovalent-ion-selective membranes for reverse electrodialysis. J. Membr. Sci..

[22] Hong J.G., Chen Y. (2015). Evaluation of electrochemical properties and reverse electrodialysis performance for porous cation exchange membranes with sulfate-functionalized iron oxide. J. Membr. Sci..

[23] Lee Y.J., Cha M.S., Oh S.-G., So S., Kim T.-H., Ryoo W.S., Hong Y.T., Lee J.Y. (2019). Reinforced anion exchange membrane based on thermal cross-linking method with outstanding cell performance for reverse electrodialysis. RSC Adv..

[24] Yang S., Choi Y.-W., Choi J., Jeong N., Kim H., Jeong H., Byeon S.Y., Yoon H., Kim Y.H. (2019). Green fabrication of pore-filling anion exchange membranes using R2R processing. J. Membr. Sci..

[25] Yang S., Choi Y.-W., Choi J., Jeong N., Kim H., Nam J.-Y., Jeong H. (2019). R2R Fabrication of pore-filling cation-exchange membranes via one-time impregnation and their application in reverse electrodialysis. ACS Sustain. Chem. Eng..

[26] Moreno J., Grasman S., van Engelen R., Nijmeijer K. (2018). Upscaling Reverse Electrodialysis. Environ. Sci. Technol..

[27] Tedesco M., Scalici C., Vaccari D., Cipollina A., Tamburini A., Micale G. (2016). Performance of the first reverse electrodialysis pilot plant for power production from saline waters and concentrated brines. J. Membr. Sci..

[28] Han J.-H., Jeong H., Hwang K.S., Kim C.-S., Jeong N., Yang S. (2020). Asymmetrical electrode system for stable operation of a large-scale reverse electrodialysis (RED) system. Environ. Sci. Wat. Res. Technol..

[29] Veerman J., Saakes M., Metz S.J., Harmsen G.J. (2010). Electrical power from sea and river water by reverse electrodialysis: A first step from the laboratory to a real power plant. Environ. Sci. Technol..

[30] Tedesco M., Cipollina A., Tamburini A., Micale G. (2017). Towards 1 kW power production in a reverse electrodialysis pilot plant with saline waters and concentrated brines. J. Membr. Sci..

[31] Choi J., Yang S., Jeong N.-J., Kim H., Kim W.-S. (2018). Fabrication of an Anion-Exchange Membrane by Pore-Filling Using Catechol–1,4-Diazabicyclo-[2,2,2]octane Coating and Its Application to Reverse Electrodialysis. Langmuir.

[32] Yang S., Kim W.-S., Choi J., Choi Y.-W., Jeong N., Kim H., Nam J.-Y., Jeong H., Kim Y.H. (2019). Fabrication of photocured anion-exchange membranes using water-soluble siloxane resins as cross-linking agents and their application in reverse electrodialysis. J. Membr. Sci..

[33] Lee M.-S., Kim H.-K., Kim C.-S., Suh H.-Y., Nahm K.-S., Choi Y.-W. (2017). Thin Pore-Filled Ion Exchange Membranes for High Power Density in Reverse Electrodialysis: Effects of Structure on Resistance, Stability, and Ion Selectivity. ChemistrySelect.

[34] Vermaas D.A., Saakes M., Nijmeijer K. (2011). Doubled power density from salinity gradients at reduced intermembrane distance. Environ. Sci. Technol..

[35] Vermaas D.A., Veerman J., Yip N.Y., Elimelech M., Saakes M., Nijmeijer K. (2013). High Efficiency in Energy Generation from Salinity Gradients with Reverse Electrodialysis. ACS Sustain. Chem. Eng..

[36] Pawlowski S., Crespo J.G., Velizarov S. (2014). Pressure drop in reverse electrodialysis: Experimental and modeling studies for stacks with variable number of cell pairs. J. Membr. Sci..

[37] Long R., Li B., Liu Z., Liu W. (2018). Reverse electrodialysis: Modelling and performance analysis based on multi-objective optimization. Energy.

[38] Moya A. (2017). A Nernst-Planck analysis on the contributions of the ionic transport in permeable ion-exchange membranes to the open circuit voltage and the membrane resistance in reverse electrodialysis stacks. Electrochim. Acta.

[39] Tedesco M., Hamelers H., Biesheuvel P. (2016). Nernst-Planck transport theory for (reverse) electrodialysis: I. Effect of co-ion transport through the membranes. J. Membr. Sci..

[40] Tedesco M., Hamelers H., Biesheuvel P. (2018). Nernst-Planck transport theory for (reverse) electrodialysis: III. Optimal membrane thickness for enhanced process performance. J. Membr. Sci..

[41] Lee M.-S., Kim T., Park S.-H., Kim C.-S., Choi Y.-W. (2012). A highly durable cross-linked hydroxide ion conducting pore-filling membrane. J. Mater. Chem..

[42] Kang H.-G., Lee M.-S., Sim W.-J., Yang T.-H., Shin K.-H., Shul Y.-G., Choi Y.-W. (2014). Effect of number of cross-linkable sites on proton conducting, pore-filling membranes. J. Membr. Sci..

[43] Kim H., Jeong N., Yang S., Choi J., Lee M.-S., Nam J.-Y., Jwa E., Kim B., Ryu K.-S., Choi Y.-W. (2019). Nernst–Planck analysis of reverse-electrodialysis with the thin-composite pore-filling membranes and its upscaling potential. Water Res..

[44] Ortiz-Martínez V.M., Gómez-Coma L., Tristán C., Pérez G., Fallanza M., Ortiz A., Ibáñez R., Ortiz I. (2020). A comprehensive study on the effects of operation variables on reverse electrodialysis performance. Desalination.

