# Supplementary material for: Correlations between Properties of Pore-Filling Ion Exchange Membranes and Performance of a Reverse Electrodialysis Stack for High Power Density
Source: Membranes (Basel). 2021 Aug 10;11(8):609. doi: 10.3390/membranes11080609 (PMC8400206; doi:10.3390/membranes11080609)
Supplement: Supplementary file 1 [file membranes-11-00609-s001.zip › membranes-1325277-supplementary.pdf]

membranes

MDPI

Supplementary Materials

Correlations between Properties of Pore-Filling Ion Exchange Membranes and Performance of a Reverse Electrodialysis Stack for High Power Density

Hanki Kim [1], Jiyeon Choi [1], Namjo Jeong [1], Yeon-Gil Jung [2,3], Haeun Kim [2,3], Donghyun Kim [2,3] and SeungCheol Yang [2,3,*]

1 Jeju Global Research Center, Korea Institute of Energy Research, Jeju-si 63357, Korea; hankikim@kier.re.kr (H.K.); jychoi@kier.re.kr (J.C.); njjeong@kier.re.kr (N.J.)
2 School of Materials Science and Engineering, Changwon National University, Changwon-si 51140, Korea; jungyg@changwon.ac.kr (Y.-G.J.); rlagkdms324@changwon.ac.kr (H.K.); 20165096@changwon.ac.kr (D.K.)
3 Department of Materials Convergence and System Engineering, Changwon National University, Changwon-si 51140, Korea
* Correspondence: pure5258@changwon.ac.kr; Tel.: +82-55-213-3715

1. Characterization of Pore-Filling Ion Exchange Membranes (PIEMs)

The ion exchange capacities (IECs) of the PCEMs and PAEMs were measured by the following method. The PCEMs and PAEMs were soaked in an aqueous 2M HCl solution and an aqueous 1M NaCl solution, respectively, for 24 h for acidification of sulfonic groups in the PCEMs, and conversion of ammonium groups into Cl^- in the PAEMs. After that, the PCEMs and PAEMs were immersed in an aqueous 3M NaCl solution and an aqueous 0.5M Na_2CO_3 solution of 50 mL for 24 h, respectively. Subsequently, the solutions from the PCEMs and PAEMs were titrated with an aqueous 0.01 M NaOH solution and an aqueous 0.01M $AgNO_3$ solution, respectively, by using an auto-titrator (848 Titrino Plus, Metrohm, Switzerland), and the IECs were calculated using the following equation:

$$\text{IEC (milliequivalents per gram (meq/g))} = (C \times V) / M_{dry}$$

Here, C is the concentration of titration solutions (0.01 M), V is the volume of the titration solutions added at the equivalence point (mL), and M_{dry} is the weight of the dried PCEMs and PAEMs.

The measurement on the PCEMs and PAEMs was performed by the following method, the PCEMs and PAEMs were immersed in an aqueous NaCl solution with 0.5M concentration for 24 h. And then, PCEMs and PAEMs were assembled by insertion between a two-compartment cell with a 1-cm^2 Pt electrode. The total resistance of the PCEMs (or PAEMs) and aqueous 0.5M NaCl solution was measured using an inductance–capacitance–resistance–impedance (LCRZ) meter (DU-6011, Delta United Instrument, Taiwan) at a frequency of 1 kHz. (R_1) In addition, a resistance of only aqueous 0.5M NaCl solution was measured. (R_2) The membrane resistances (R) were calculated using the following equation.

$$R = (R_1 - R_2) \times S$$

Here, S is the effective membrane area.

The permselectivities of the PCEMs and PAEMs were measured by the following method. First of all, all membranes were soaked in an aqueous 0.017M NaCl solution for 24 h. And then, the PCEMs and PAEMs were assembled by insertion between a two-compartment cell. Each compartment in the two-compartment was filled with aqueous 0.5M and 0.017M NaCl solutions. During the membrane potential (E_m) measurements using

digital multimeter and two Ag/AgCl reference electrodes, aqueous 0.5M and 0.017M NaCl solutions were stirred to minimize the boundary-layer effects.

The transport number of counter-ions (t) in each PIEM was obtained using the membrane potential and the following equation.

$$\mathbf{E_m = (2t - 1)\frac{RT}{zF}\ln\frac{a_1}{a_2}}$$

Here, t, T, R, F, z, a_1, and a_2 are the transport number of cations and anions in the PCEM and PAEM, respectively, absolute temperature, gas constant, Faraday constant, counter-ion electrovalence, the activity of the aqueous 0.5M NaCl solution, and the activity of the aqueous 0.017M NaCl solution, respectively.

The permselectivity of the PCEM and PAEM was obtained using the following equation.

$$\boldsymbol{\alpha} = \frac{\mathbf{t_M^m - t_M^s}}{\mathbf{t_X^s}}$$

Here, α and t is the permselectivity and transport number, respectively. Subscripts M and X mean counter- and co-ions, respectively. Superscripts m and s indicate the membrane and the solution phases, respectively.

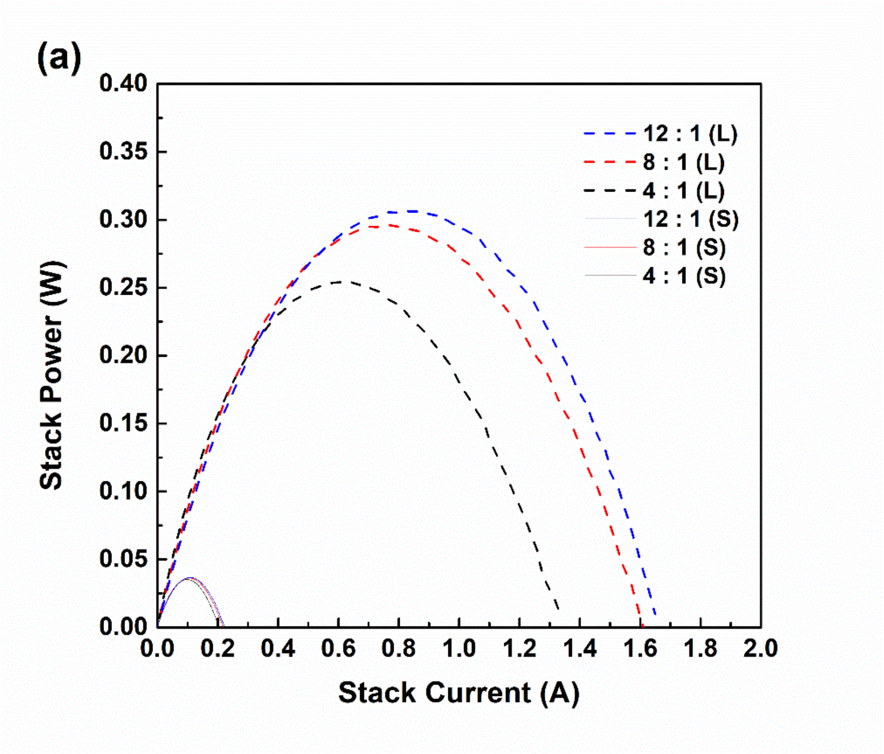

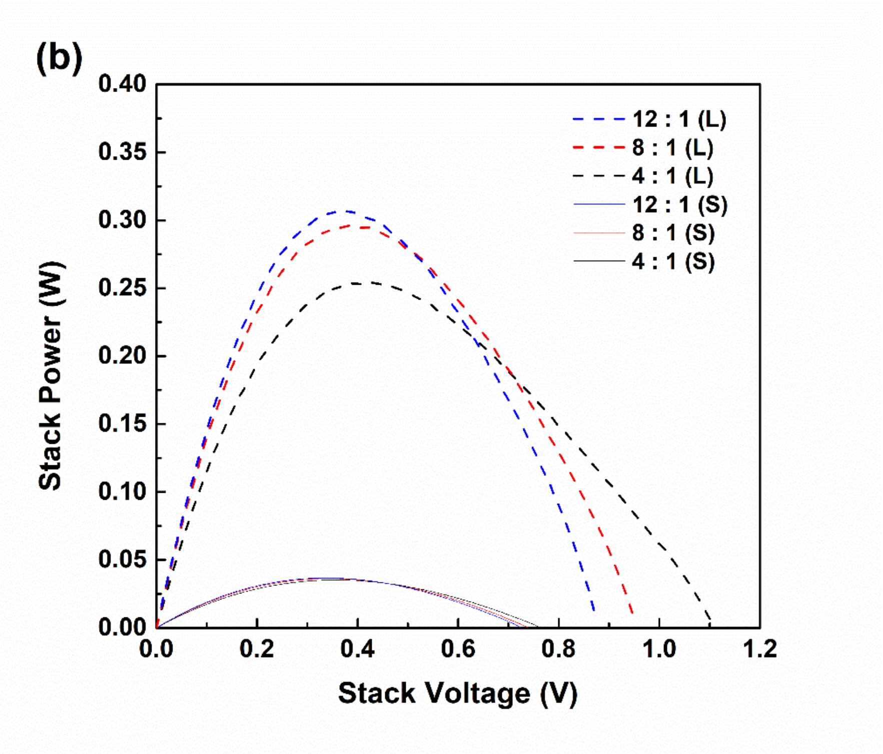

Figure S1. (a) Stack current-power curves, and (b) stack voltage-power curves according to a composition of PIEMs and size of RED stacks. “S” and “L” in Figure indicates results obtained from small and large RED stack, respectively.
